# Air-Assisted Sprayed Flexible Cellulose Acetate/Chitosan Materials for Food Packaging

**DOI:** 10.3390/polym17182479

**Published:** 2025-09-13

**Authors:** Nasrin Moshfeghi Far, Ana Kramar, Javier González-Benito

**Affiliations:** 1 Department of Materials Science and Engineering and Chemical Engineering, Universidad Carlos III de Madrid, Av. Universidad 30, 28911 Leganés, Spain; 2 Novel Materials and Nanotechnology Group, Institute of Agrochemistry and Food Technology (IATA), Spanish Council for Scientific Research (CSIC), Calle Catedrático Agustín Escardino Benlloch 7, 46980 Paterna, Spain; akramar@iata.csic.es

**Keywords:** food packaging, cellulose acetate, chitosan, air-assisted solution spraying, solution blow spinning, multifunctional materials

## Abstract

Cellulose and chitin are the most abundant natural polymers, and their exploitation paves the way for sustainable materials and products. This work investigates the preparation of composites based on acetylated cellulose and partially deacetylated chitin, i.e., chitosan, using versatile and robust air-assisted solution spraying (AASS), a potential method for preparing materials both in situ and ex situ. These materials, in the form of films, despite being prepared from high-molecular-weight and rigid biopolymers, show high flexibility (Young’s moduli below 1 GPa), outstanding mechanical properties (tensile strengths above 19 MPa and strain at failure higher than 2%), and bioactivity towards *E. coli*. The unprecedented flexibility, obtained without the use of any plasticizer or by casting with humidity control, is a direct consequence of the specific film morphology, whereby films are constituted from merging droplets. Depending on the solution properties (viscosity, surface tension), various droplet sizes are obtained, thus influencing the roughness and indirectly the wettability. Wettability analysis towards water and oil revealed higher contact angles towards both fluids as the content of chitosan increases in the composite what directly impacts packaging applications by better protecting the food. Besides this, higher chitosan content in the composite (7.5% *w*/*w*) enabled bioactivity against *E. coli*, where colony development was inhibited on the film surface compared with the neat cellulose acetate. This study shows a very high potential for AASS for obtaining uniform thin flexible films for food packaging applications, allowing faster drying and lower energy consumption than other film-forming techniques.

## 1. Introduction

There is growing awareness in society of the fundamental danger lying in the non-biodegradability of plastic materials, which accumulates in the environment, causing negative impacts [1]. As a result, the use of sustainable bioplastics has gained significant interest, mainly in sectors such as food packaging. Among these materials, cellulose acetate (CA) is receiving special attention because it arises from cellulose, which is one of the most abundant polymers on Earth, and it has a high ability for film and fiber formation [2,3]. Furthermore, CA can have interesting properties when it is constituted by nanofibers, particularly with the advent of electrospinning. Among others, cellulose acetate (CA) nanofibers find several applications, including tissue engineering, filtration membranes, and wound dressings [4,5,6]. In the particular case of food packaging, electrospinning and solution casting have been recently revisited for CA, allowing the preparation of thin materials suitable for use as polymeric films for food packaging [7,8]. Besides its natural origin, being the derivative of cellulose, cellulose acetate is convenient from a circular bioeconomy aspect, as it is biodegradable as well. Although without directly using the ASTM D6400 specification Juergen Puls et al. used the experimental conditions (aerobic composting and CO_2_ evaluation using standard methods) to report that CA films with a DS = 2.57 require about 18 days to fully degrade under composting conditions, using a Sturm-type test (based on CO_2_ evolution measurement) [9]. CA of lower DS (below 2) is readily biodegradable under appropriate composting conditions, but is also relatively hydrophilic. Therefore, it is important to find adequate biodegradability, a balance between hydrophilicity and hydrophobicity, while maintaining good mechanical properties and flexibility at the same time, for food packaging applications.

However, achieving this balance of properties can be challenging, especially when using hydrophilic and rigid polymers like polysaccharides, such as cellulose acetate (CA). Their rigidity, due to their intra- and intermolecular interactions (especially hydrogen bonds in partially hydroxylated regions), can lead to brittleness if they are not conveniently modified with plasticizers or prepared under specific relative humidity [10]. Food quality and safety quickly deteriorate when made from hydrophilic materials because their water barrier properties are poor [11]. Although CA film exhibits a static water contact angle of approximately 60°, indicating that it is hydrophilic, but less so than pure cellulose [12], representing a little improvement with respect to neat cellulose. However, it would be interesting to achieve an even lower hydrophilicity. In this sense, several approaches based on modifications with other polymers and particles have been tried, for example, using polyvinylidene fluoride (PVDF) [13] or PVDF with poly methylmethacrylate (PMMA) [14] in the case of polymers, and among others, SiO_2_, ZnO as nanoparticles [15]. Besides modifying CA with other components, sometimes a better way to tune hydrophobicity is to tailor the morphology of the materials (size and shape of nano or microfeatures, pores, for instance) as has been demonstrated by the use of electrospinning to achieve hydrophobic and even superhydrophobic properties [16,17]. It is clear, therefore, that many factors influence the final wettability behavior of these materials, and further research should be conducted to shed more light on this issue. In terms of the optimization of morphology, processing conditions constitute a very important issue to take into account. Electrospinning (ES) is perhaps the most well-known and currently used technique to obtain complex morphologies from continuous films to nanofibrous polymer-based materials [18]. However, some drawbacks can be considered for its use; for example, a high electric field is required, there is a limitation in the choice of solvents for polymers to those that interact properly with the electric field, and the production rates are relatively low. For this reason, other techniques should be explored to attempt to overcome the aforementioned drawbacks. Among possible techniques, solution blow spinning (SBS), or gas-assisted solution spraying when fibers are not obtained, is being studied to address those limitations [19,20]. In terms of morphologies, SBS offers results comparable to electrospinning but with a faster production rate, using a pressurized gas instead of an electric field as the driving force to eject and stretch the solution and deposit the polymer-based material onto a substrate. In addition, SBS is industrially scalable by using multi-nozzle or continuous spray heads mounted on automated lines where the so-produced atomized or spun polymer must be deposited onto a moving substrate or conveyor belt [21].

In addition, SBS, or more conveniently and from now on called air-assisted solution spraying (AASS), when air is used as the gas and no fibers are obtained, was already used to prepare films for different purposes [22,23], which reinforces the idea of preparing CA-based materials through the use of SBS. Among the advantages offered by AASS, it can be highlighted that there is the possibility of dispensing the materials over any kind of substrate, allowing the “in situ” preparation of materials. Considering the above-mentioned, this processing method would be highly versatile since it allows those materials to be directly sprayed on foods, fruits, for instance, or simply deposited on a substrate from which they would be extracted for other purposes, such as the preparation of functional sachets for food packaging applications.

However, AASS is proving challenging for the processing of CA [22], because of the difficulty in producing very particular morphologies such as defect-free nanofibers. CA spinnability by AASS can be facilitated by the use of other polymers included in the spun solution. According to Claro and coworkers, poly(ethylene oxide) (PEO) was used together with CA to finally produce fibrous CA-based materials by AASS in the form of flexible ionic conductive cellulose mats [24]. It was also successfully produced CA fibers using PEO as a spinning aid, showing the importance of the molecular weight of polymers and solvents [22]. By co-spinning CA nanofibers with polyacrylonitrile, Dadol and coauthors also successfully produced CA nanofibers [25]. Therefore, still more efforts should be focused on finding easier AASS conditions to prepare nanofibrous CA-based materials. Therefore, looking for adequate processing conditions and a solution system, nearly any morphology should be able to be obtained.

On the other hand, plastic materials for food packaging should possess additional properties such as mechanical consistency, chemical resistance, thermal stability, and environmental friendliness, most of which are already provided by CA. However, it is highly recommended to incorporate special functionalities, such as antimicrobial activity and the ability to delay oxidation processes.

The most common way to impart antibacterial activity to plastics for food packaging is by the incorporation of antimicrobial agents, such as natural organic compounds like nisin [26], chitosan [27], and essential oils [28] or inorganic nanoparticles like silver (Ag) nanoparticles [29], zinc oxide (ZnO) nanoparticles [30] and titanium dioxide (TiO_2_) nanoparticles [31].

Among them, chitosan (CS) is currently receiving special attention due to multiple reasons that make it very attractive for food packaging purposes. CS has a natural origin, is biodegradable, and non-toxic. CS is derived from chitin that can be extracted from shrimps and crab shells, making it a sustainable and eco-friendly material. CS is recognized as GRAS (Generally Recognized as Safe) by the FDA for food applications, without releasing harmful residues. In addition, CS has a broad spectrum for antimicrobial activity since it is effective against Gram-positive and Gram-negative bacteria, fungi [32], and even some viruses [33,34]; however, the antiviral effectiveness of chitosan is still not well understood and depends on factors such as pH, charge, molecular weight, etc. CS can enhance oxygen and moisture barrier properties, prolonging shelf life [35,36], and finally, CS has shown great potential for controlled release processes of antimicrobial compounds to extend food preservation [37]. The antimicrobial activity of chitosan itself mostly depends on the degree of substitution (deacetylation) and molecular weight.

The study focuses on optimizing the air-assisted solution spraying (AASS) as a potential sustainable, effective, and low-cost processing method to prepare new materials for food packaging. These materials are prepared in the form of films mainly constituted by cellulose acetate (CA) with improved properties in terms of flexibility, antimicrobial action, and hydrophobicity, for a better performance as a food packaging material. In this sense, a full characterization of the films’ morphology, mechanical strength, and wettability, together with the evaluation of their antibacterial activity against E. coli, is carried out. To tailor the surface properties and enhance the final performance of materials for food packaging and enhance food shelf life, CA is modified by the addition of chitosan (CS), and the morphology of the materials is controlled by selecting appropriate conditions of the AASS processing method (pressurized air flow, solution feeding rate, working distance, and composition of polymer solutions) for preparation of thin film-like materials from a polymeric blend. Therefore, this work aims to develop innovative films from cellulose acetate and chitosan using AASS, without plasticizers, suitable for food packaging applications and aligned with environmental sustainability goals.

## 2. Materials and Methods

### 2.1. Materials

Cellulose acetate, CA (Sigma Aldrich, St. Louis, MO, USA, average Mn ~ 30,000 g/mol, acetyl content 39.8 wt%, degree of substitution, DS = 2.45 that represents the average number of acetate groups per anhydroglucose unit) and chitosan, CS, (Sigma-Aldrich, St. Louis, MO, USA, low molecular weight, zero-shear viscosity measured using Haake Viscotester IQ–Thermo Fisher Scientific (Waltham, MA, USA), at 25 °C of 1 wt% solution in 2% acetic acid is 1.73 Pa·s, degree of deacetylation, DDA = 66%, according to the method described elsewhere [38]) were used as received. After a deep solubility study (see Supplementary Materials), it was found that the best solvent systems for this set of polymers were formic acid, FA (Panreac, Barcelona, Spain, 85% purity), and acetic acid, HAc (Panreac, Barcelona, Spain, glacial 91% purity). The use of 91% acetic acid is intended to improve the spray processing of the corresponding polymer solution. Since the solvents are not involved in a chemical synthesis and are ultimately removed during the AASS preparation of these materials, the choice of 91% acetic acid is considered acceptable, particularly as it is expected to facilitate solvent evaporation during the AASS process.

As a pH indicator for the titration of CA to obtain the degree of substitution DS, Phenolphthalein (Panreac, Barcelona, Spain, 318.33 g/mol) was used. On the other hand, methyl orange (Panreac, Barcelona, Spain, 327.34 g/mol) was used as the pH indicator for the titration of CS to obtain its degree of deacetylation, DDA. Ethanol, EtOH (Sigma Aldrich, St. Louis, MO, USA, 96% purity), was used to dissolve CA. Aqueous solution of sodium hydroxide 1.0 M, NaOH (Sigma Aldrich, St. Louis, MO, USA), was used for saponification of CA and back-titration of CS, while standardized hydrochloric acid solution 1.0 M, HCl (Sigma Aldrich, St. Louis, MO, USA), was used for back-titration of CA and initial dissolution of CS.

### 2.2. Degree of Substitution (DS) of CA

To understand the properties and behavior of materials prepared in this work, information about the degree of substitution (DS) of CA is required, or the average number of acetyl groups substituting the hydroxyl groups within the glycosidic units. A typical method of saponification with subsequent back-titration was carried out. The process was performed in triplicate at room temperature, with continuous magnetic stirring for 60 min to obtain both the degree of acetylation (%*AG*) and DS by the use of Equations (1) and (2) that were adapted from Samios et al. [38], where the factor 43 g/mol refers to the molar mass of the acetyl group (CH_3_CO–) and the value 102.40 represents the molar mass of the original repeating unit (before acetylation) used in the calculation of substituents.

(1) $$\%AG=\frac{[\left(V_{b_{i}}-V_{b_{t}})\times C_{b}-V_{a}\times C_{a}\right]\times 43\times 100}{m_{CA}}$$

(2) $$DS=\frac{3.86\times \%AG}{102.40-\%AG}$$

where $V_{b_{i}}$ is the volume of NaOH added to the system, $V_{b_{t}}$ is the volume of NaOH utilized for titration, *C_b_* is the molar concentration of the base, *V_a_* is the volume of HCl introduced into the system, *C_a_* is the molar concentration of the acid, and *m_CA_* is the mass of CA used.

### 2.3. Degree of Deacetylation (DDA) of CS

Similarly to CA, the degree of deacetylation of CS is required to better understand the final properties. Also, in this case, an acid-base titration was used following the method described in [39] and using Equation (3).

(3) $$DDA=\left[1-\frac{\left(V_{b}\times C_{b}\times 161\right)}{\left(m_{CS}\times 1000\right)}\right]\times 100$$

where *V_b_* is the volume of NaOH (in mL) used for the titration, *C_b_* is the concentration of NaOH used in mol/L, 161 is the molar mass of the glucosamine monomer unit (C_6_H_11_NO_4_), in g/mol, and *m_CS_* is the weight in grams of CS used.

### 2.4. Sample Preparation

Before choosing the best conditions to obtain solutions where both CA and CS are compatible with a solvent system, a deep solubility study was performed (see Supplementary Materials). Solvents and solubility conditions were chosen considering the most homogeneous stable solutions in terms of clarity or liquid transparency, for which no phase separation was considered.

First, the polymer solutions to be blow spun were prepared by dissolving the polymer systems in both FA and HAc. CA was added to the corresponding solvent to reach a concentration of 8% by weight. Then, specific amounts of CS were added to prepare the final solid materials with different compositions for the blend CA/CS, 0%, 2.5%, 5.0% and 7.5% by weight in chitosan (percentages correspond to the relative amount of chitosan with respect to the whole blend or material), which were labeled as CA, CA/CS-1; CA/CS-2 and CA/CS-3, respectively, adding the prefix HAc- or FA- depending on the solvent used.

Once the solutions are prepared, they are subjected to AASS using a home-designed and made automated device [40]. Through the use of an automatic pump and a syringe, solutions are injected in a coaxial nozzle with an inner channel of 0.6 mm diameter protruding 2 mm from the nozzle. On the other hand, pressurized air is passed through the outer nozzle of 1.0 mm, exerting the required force on the solution to spray it to the collector while favoring the evaporation of solvent. The materials were collected on a rotating drum (rotational speed 250 rpm). When the solvent used was HAc, AASS processing conditions were 12 cm of working distance (WD) or length between the collector and the inner nozzle, solution injection rate of 0.125 mL/min, and air pressure of 1.0 bar. When the solvent used was FA, AASS processing conditions were 15 cm of WD, a solution injection rate of 0.125 mL/min, and an air pressure of 1.5 bar. The AASS conditions, chosen according to the solvent, were selected simply through several trial-and-error tests until those that ensured the formation of a homogeneous film in terms of thickness and prevented nozzle clogging during the AASS process were identified. As an example, in Figure S1, representative images of films obtained by spraying are shown (regardless of the composition and the solvent used, all films are very similar), for which similar thicknesses were obtained, ~300 µm.

### 2.5. Methods and Equipment

#### 2.5.1. Viscosity

Viscosity measurements were performed using a Haake iQ viscometer. Solutions or suspensions were placed between two circular plates at constant temperature (25 °C), and subjected to an oscillatory shear force, in the frequency range 100–1000 s^−1^, with an acquisition time of 30 s per measurement (100 points). In Table 1, the values of viscosities in Pa·s obtained at 0.1 kHz for all systems to be sprayed are collected.

#### 2.5.2. Surface Tension

Surface tension was determined using the pendant drop method with a tensiometer OCA-15 Plus Goniometer (Data Physics, Neuterk Instruments, Eibar, Spain). A standard needle with a diameter of 1.65 mm was used, and at least 10 drops were measured per sample, with photographs taken 30 s after the drop formation at room temperature. Finally, from the pear-shaped drop image analysis and using the Young-Laplace equation, the surface tension was obtained. In Table 1, the values of surface tensions in mN·m^−1^ of all systems prepared to be sprayed are collected.

One-way ANOVA test showed there is a statistically significant difference (with α level 0.05) in the values of viscosity and surface tension between the solutions that contain different amounts of chitosan.

#### 2.5.3. Structural Characterization

Attenuated Total Reflectance Fourier transform infrared spectroscopy (ATR-FTIR) was used to analyze the chemical structure of materials. A Nicolet iS5 spectrometer (Thermos Scientific, Thermofisher, Madison, WI, USA) and an attenuated total reflectance (ATR) device with a diamond window GladiATR (PIKE Technologies, Madison, WI, USA) were utilized. To obtain the spectra, 32 scans at a resolution of 4 cm^−1^ within the range 400–4000 cm^−1^ were performed with a Golden Gate ATR accessory to create Fourier-transformed infrared spectra.

On the other hand, X-ray diffraction (XRD) was also used to study the structure of the materials. XRD patterns were obtained using an X-ray powder diffractometer Bruker ECO D8 Advance (Bruker, Karlsruhe, Germany) from the interaction with the Cu-Kα1 radiation (λ = 1.5418 Å) in the range 5° to 50° in 2θ, with a scanning rate of 2 s per step and a step size of 0.02°.

#### 2.5.4. Morphological Characterization

The morphology of the materials was inspected by field emission scanning electron microscopy (FESEM) using a TENEO-FEI field emission scanning electron microscope. FESEM images were generated from secondary electrons from a primary electron beam accelerated with a 10 kV potential and detected with an ETD detector. Samples were mounted onto SEM stubs using double-sided carbon tape and then sputter-coated with gold for 90 s using a Leica EM ACE200 low-vacuum coater (Leica Microsystems, Wetzlar, Germany) to render them conductive, preventing both electrostatic charge buildup and localized heating. To analyze images and determine different morphological parameters, the free software ImageJ (V.1.52a, National Institutes of Health, Bethesda, MD, USA) was used.

The morphology was also studied using an Olympus DSX500 optical profilometer (Olympus Iberia, Barcelona, Spain). In this case, images were used, among other things, to determine roughness parameters using the cut-off wavelength, *λ_c_*, or the length scale used to filter out surface features larger or smaller than it when measuring roughness, ensuring that only the relevant roughness is analyzed according to ISO 4288 [41].

Regardless of the technique used to study the morphology, only the surface of the samples directly in contact with air during their preparation by AASS was inspected.

To determine the porosity, the well-known gravimetric method was used [42]. Specimens of samples were weighed out in an analytical balance to obtain the mass, m, and dimensioned using an Easy-check Neurtek Instrument with an accuracy of ±1 μm to measure thicknesses, h, and a digital caliper to measure widths, w, and lengths, l. In this way, volumes of specimens were obtained as *h* × *w* × *l*. Finally, the specimen density was obtained by dividing the mass by the volume. After obtaining the densities of three specimens, the final sample density was calculated as an average (*ρ_s_*). The porosity was finally determined following Equation (4), where theoretical density (*ρ_t_*) is included, and that was estimated from a simple rule of mixtures (Equation (5)).

(4) $$\phi =1-\frac{\rho_{s}}{\rho_{t}}$$

(5) $$\rho_{t}=\rho_{CA}\cdot \phi_{CA}+\rho_{CS}\cdot \phi_{CS}$$

where *ρ_AC_* and *ρ_CS_* are the densities of the polymers, cellulose acetate (~1.3 g·cm^−3^ [43]) and chitosan (~1.5 g·cm^−3^ based on the data reported by D. Lupa et al. [44]), respectively, and *ϕ_AC_* and *ϕ_CS_* are the volume fractions in the corresponding sample.

#### 2.5.5. Water Vapor Permeability

To study the water vapor permeability, the water vapor transmission rate (WVTR) was determined using the method provided in the standard ISO 2528:2017 [45], slightly modified to test the kind of specimens prepared in this work. The experiments were conducted in triplicate, and the mass of water per square meter of film per day was utilized to compute the permeability.

#### 2.5.6. Solvophilicity Behavior

Water and oil contact angle measurements were performed in an OCA-15 Plus Goniometer (Data Physics, Neurtek Instruments, Eibar, Spain) through the sessile drop contact angle method. Photographs of 3 µL volume of distilled and deionized water or oil (extra virgin olive oil) drops deposited on the films were captured immediately after their deposition. After that, image analysis was carried out to obtain the angle formed between the droplet and the surface of the material. At least five measured angles were used to finally obtain the contact angle as an average. It is important to highlight that surface tension (mN·m^−1^) and surface energy (mJ·m^−2^) are numerically equivalent and represent the same quantity.

#### 2.5.7. Thermal Behavior

Thermal relaxations of the films were studied by differential scanning calorimetry (DSC) using a Mettler Toledo 822e calorimeter under a nitrogen atmosphere. Samples (4–6 mg) were weighed in aluminum pans sealed with perforated covers to keep constant pressure. After that, samples were subjected to three thermal cycles of heating-cooling-heating, only the first heating was used to compare the thermal behaviors of the different materials, since information from the prepared materials is required for the present work. This first heating cycle consisted of a ramp of temperatures from 50 °C to 260 °C at 10 °C/min. Glass transition and melting temperatures were extracted from the thermograms, and the crystalline fraction of the materials, *χ*, was calculated using Equation (6) from the area of the endothermic peak and dividing by the corresponding enthalpy of fusion of triacetate cellulose 100% crystalline, Δ*H*_100_ = 58.8 J·g^−1^ [46].

(6) $$\chi =\frac{∆H_{m}\cdot (1-x)}{∆H_{100}}$$

where Δ*H_m_* is the enthalpy of fusion obtained from the area under the endotherm of the first heating cycle, and *x* is the weight fraction of chitosan.

#### 2.5.8. Mechanical Behavior

Tensile tests of samples were carried out using a universal testing machine, Microtest DT/005/FR (Microtest S.A., Madrid, Spain) with a load cell of 50 N. Rectangular specimens (40 mm length, 10 mm width, and ~0.02 mm thickness) were tested, using a gauge length of 20 mm and applying a loading rate of 5 mm/min. At least 5 specimens (cut following the direction of collector rotation) of each sample were tested to obtain, from the analysis of the corresponding stress–strain curves, the mechanical parameters as mean values. Before each test, specimen thickness was measured using a Digimatic micrometer (Mitutoyo Corporation, Barcelona, Spain) with ±1 μm accuracy.

#### 2.5.9. Bioactivity Assay

To ensure the absence of microorganisms, all materials were sterilized by exposure to ultraviolet (UV) light for a minimum of 20 min before use. A commonly used Escherichia coli strain, DH5α, was selected for bacterial culturing aligned with methods outlined in ISO 22196 [47] and ASTM E2149 [48]. The idea of using a DH5α strain is to begin with a preliminary and straightforward study of the antimicrobial activity of the materials under investigation, more specifically, the effect of the presence of chitosan. To prepare the bacterial suspension, 90 μL of bacterial culture was mixed with 2910 μL of Luria–Bertani (LB) medium, resulting in a total volume of 3 mL. The mixture was incubated overnight at 37 °C with continuous shaking to promote bacterial growth. On the second day, the overnight bacterial culture was diluted 1:100 in fresh LB medium. Subsequently, 1 mL of this diluted bacterial suspension was added to each test material. The samples were then incubated at 37 °C for 3 h with shaking to allow bacterial adhesion. Following the incubation period, all liquid was removed, and the samples were washed by adding 1 mL of physiological saline solution (0.9% NaCl). The solution was gently agitated and subsequently discarded to remove excess bacteria. To collect the bacteria adhered to the surface of the test materials, sterile cotton swabs were used. The swabs were gently pressed against the surface of the samples to ensure bacterial collection, and then they were immersed in fresh LB medium. This mixture was incubated at 37 °C for 1 h to allow bacterial resuspension in the liquid. After incubation, serial dilution and plating were followed. The cotton swabs were removed, leaving a liquid containing the collected bacteria. Serial dilutions were performed in a stepwise manner: 1/100 (10^−2^); 1/1000 (10^−3^); 1/10,000 (10^−4^) and 1/100,000 (10^−5^). An LB-agar plate was divided into four sections, and 10 μL of each dilution was carefully placed onto the corresponding section. The plate was incubated overnight at 37 °C in an inverted position to facilitate bacterial colony formation. This method allows for the evaluation of bacterial attachment and viability on different materials, providing a quantitative assessment of their antibacterial properties.

#### 2.5.10. Statistical Analysis

When enough information was available to conclude, to determine whether there are statistically significant differences between the means of eight independent groups of data (associated with parameters of characterization) corresponding to eight samples studied, One-Way ANOVA analysis of variance was performed with a significance level of 0.05.

## 3. Results

### 3.1. Structure of the Materials

XRD analysis was performed to study possible changes in the polymer structure as a result of the AASS process and interactions between polymers in the blends. Figure S2 depicts the X-ray diffraction patterns of the as-received polymers CA and CS, respectively. In the case of as-received CA, three sharp peaks at 9°, 10°, and 13° are observed, which correspond to residual crystalline domains from cellulose [49]. On the other hand, peaks at 17°, 21°, and 23° of crystallographic planes (110), (020), and (002) reveal the presence of typical cellulose acetate [50]. When analyzing the CS diffractogram, it is observed that the typical peaks at 10° and 20° are associated with the (020) and (110) planes [51].

**Figure 1:**
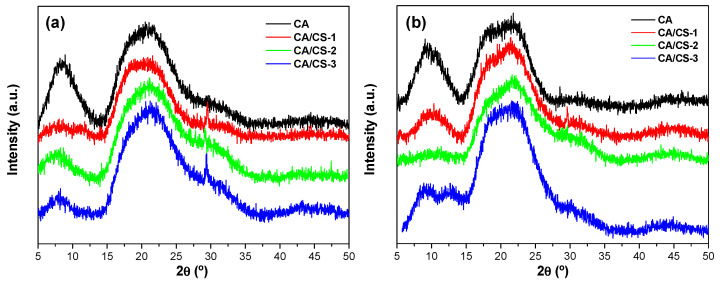
XRD patterns of the different systems prepared by AASS based on CA. (**a**) Acetic acid (HAc) and (**b**) Formic acid (FA) used as solvents.

In Figure 1, the XRD patterns of the different systems prepared by SBS based on CA are shown. Pure chitosan could not be prepared by AASS because of the difficulties of nozzle clogging. As can be seen, regardless of the solvent used to dissolve the polymers (HAc and FA), the highly ordered structure of the as-received polymers was lost, being ever more evident when chitosan is added, as can be deduced from the relative decrease in intensity of the broad peak appearing at 2θ = 8.5°. Therefore, as a conclusion of these results, it can be said that AASS processing of CA decreases its crystallinity, being further promoted by the presence of CS.

**Figure 2:**
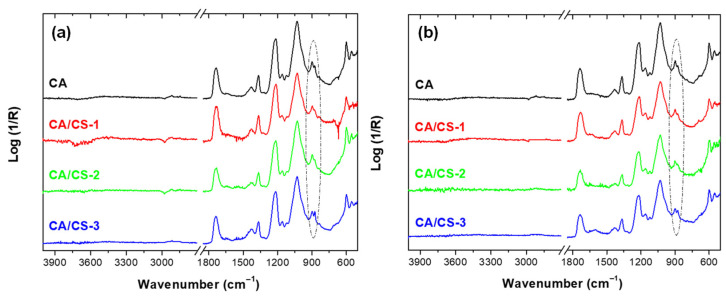
Normalized FTIR spectra of the different systems prepared by AASS based on CA. (**a**) Acetic acid (HAc) and (**b**) Formic acid (FA) used as solvents.

In order to find more information about not only the appearance of crystals, but also specific interactions between the polymers in the CA/CS blends, ATR-FTIR was utilized. Typical peaks associated with cellulose acetate were discernible across all spectra (Figure 2). The typical bands of cellulose acetate at 1737 cm^−1^ (carbonyl, C=O, stretching of the acetyl groups), at 1220 cm^−1^ (acetate ester groups, C–O), and 1030 cm^−1^ (C–O–C stretching of glycosidic linkage in cellulose backbone) [52] do not experience any change with the solvent used for AASS nor with the addition of chitosan. In addition, barely noticeable, the broad O–H stretching band at ~3470–3550 cm^−1^ corresponds to residual hydroxyl groups, which points out a high degree of substitution. With respect to chitosan, considering its low concentration in the blends, it is expected that its characteristic absorption bands will be overlapped by the CA bands. In fact, identification of specific chitosan bands, such as the N–H amine stretching band, was not possible. Upon the addition of CS significant shifts or changes in CA bands were not detected which suggests limited chemical interaction The only small difference observable in the materials obtained with both solvents is the relative increment of a small peak at 875 cm^−1^ concerning the peak at 899 cm^−1^ that can be assigned to the cellulose ring vibrations (C–O–C and C–H bending). The slight variation can only be attributed to a minor change in the crystallinity of CA, which may result from a simple nucleating effect induced by the presence of CS. In order to confirm this last observation, DSC was carried out to identify thermal transitions and determine the degree of crystallinity.

Here, it is convenient to highlight that the presence of acetic acid or formic acid in plastics can lead to rejection for food packaging applications by the FDA, depending on their concentration, purpose, and potential migration into food. However, in principle, the idea of using AASS is to achieve a material completely free of solvents. This objective is usually fulfilled thanks to the intense work performed looking for the proper processing conditions, which, for this particular issue, are related to the air pressure and the working distance. One way to ensure the absence of solvents in the final materials is through information delivered by FTIR. As can be seen in Figure 2, there is no evidence of HAc or FA, since the intense peaks corresponding to the O-H stretching vibrations that must be found in a broad band from 3200 to 3550 cm^−1^ are not observed. Therefore, in the case of the presence of these two solvents, they should be in the form of traces below the sensitivity limit of the spectrophotometer, which is around 0.5%. However, before validating the materials for their final use, a migration test using food simulants should be conducted.

**Figure 3:**
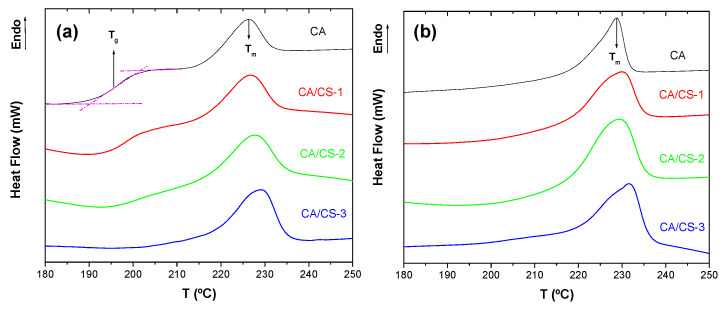
DSC thermograms are observed for the AASS CA-based materials prepared in the present work. (**a**) Acetic acid (HAc) and (**b**) Formic acid (FA) used as solvents.

In Figure 3, complex DSC thermograms are observed for the AASS CA-based materials prepared in the present work, where several endo- and exothermal phenomena are shown. This particular complex thermal behavior is found in other cellulose acetate systems [53,54], which usually has led to contradictory discussions. Due to this, the assignment should be made, being supported by results obtained by other techniques, such as DMA. Following this, and based on the results obtained by Scandola et al. [53,55], it seems more reasonable to assign the glass transition, or more accurately, the α relaxation, to the heat capacity increase observed at around 195 °C (Figure 3); however, it should ideally be confirmed by complementary techniques, such as DMA. Finally, there is a clear endothermic peak at 225 °C associated with the melting process of CA [56]. Finally, although there is some evidence in the bibliography of a small endothermic peak for chitosan between 130 °C and 150 °C, which is assigned to the relaxation of chitosan chains rather than melting [57], in our samples, it is not expected any clear signal because of the small amount of chitosan in the whole sample (maximum 7.5% wt), which would mean about 0.4 mg in the analyzed sample. In Table 2, values of glass transition temperature, *T_g_*, melting temperature, *T_m_*, and degree of crystallinity, *χ*, are gathered for the samples of CA-based materials.

As can be seen, only when samples are prepared using solutions made in acetic acid, both glass transition and fusion of CA are observed, pointing out that more crystalline samples are obtained when formic acid is used as solvent. In fact, in Table 2 values of crystallinity degree reflect that samples prepared with formic acid as solvent present around 65% higher crystallinity. A quite lower fraction of the amorphous phase must be the reason the calorimeter is not sensitive enough to show the change in the heat capacity associated with the glass transition. One possible reason for this result is the possibility of higher deacetylation of CA when it is dissolved in FA. In fact, on average, the ratio between the absorbances at 1030 cm^−1^ (C–O–C stretching of glycosidic linkage in cellulose backbone) and at 1220 cm^−1^ (acetate ester groups, C–O) is higher when acetic acid is used as the solvent (0.8 > 0.7). It is known that the higher the degree of substitution (DS) in cellulose, the lower the crystallinity. For example, cellulose acetate with a DS of 2.5 (coincident with our as-received CA) exhibits a crystallinity index close to 10% (similar to our results when CA-based materials are obtained from a solution made with HAc, Table 2), and the lower the DS, the higher the crystallinity [58]. Here, it is proposed that formic acid, being a protic and relatively strong organic acid, can act as a catalyst for the hydrolysis of the ester bonds in cellulose acetate [59]. This results in the cleavage of acetyl groups (-COCH_3_) and the regeneration of hydroxyl (-OH) groups on the cellulose backbone. However, the above reason should be taken with caution because stronger chemical evidence would be necessary, such as direct chemical analysis for the determination of the degree of substitution.

On the other hand, two behaviors are observed as a function of the concentration of CS, depending on the solvent used to prepare the solutions to be blow spun. When HAc is used as a solvent, there is an increase in crystallinity as the concentration of CS increases. This result can be explained through the consideration of a nucleation effect since CS is less soluble in HAc, so it is expected to have small CS domains within the CA matrix while the latter is solidifying because of solvent evaporation. On the other hand, when FA is used as a solvent, there is a slight decrease in the crystallinity as the concentration of CS increases. This result can be explained by partial neutralization of FA because of the presence of the amino groups, which should shift the hydrolysis of CA to the reactants or lower deacetylation. In addition, FA is a better solvent for CS than HAc.

### 3.2. Morphology

**Figure 4:**
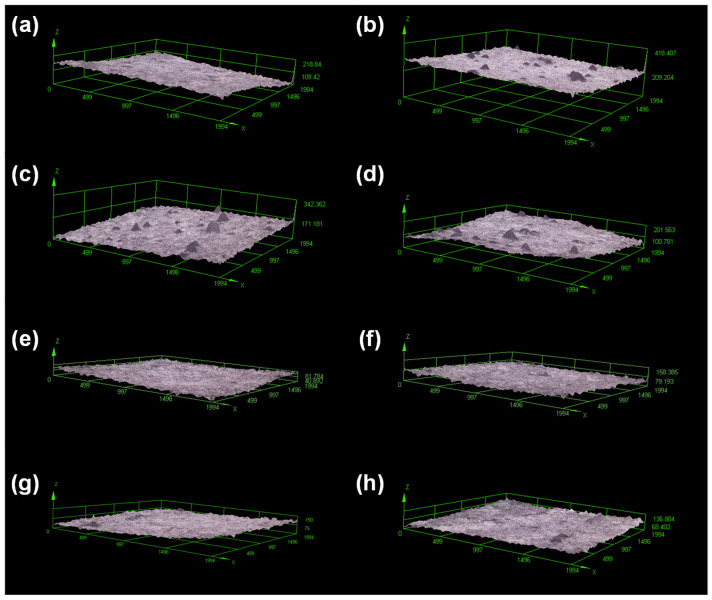
Representative 3D images obtained with an optical profilometer of the surfaces of cellulose acetate/chitosan films prepared by AASS (axes units, μm). (**a**) HAc-CA; (**b**) HAc-CA/CS-1; (**c**) HAc-CA/CS-2; (**d**) HAc-CA/CS-3; (**e**) FA-CA; (**f**) FA-CA/CS-1; (**g**) FA-CA/CS-2; (**h**) FA-CA/CS-3.

Optical profilometer and scanning electron microscopy were the microscopy techniques chosen to study the morphology of the sprayed CA-based samples. The analysis of the morphology of samples is crucial to understand several properties, such as solvophilicity or the ability of a solvent to impregnate a surface. In Figure 4, topographical images obtained with the profilometer are presented.

As can be seen, when using acetic acid as the solvent, more heterogeneous surfaces were obtained for the CA-based materials, where randomly dispersed surface small protuberances are observed over the entire surface. In addition, it seems that the higher the concentration of CS, the more heterogeneous the topography. However, it is also interesting to have quantitative topographical information from the roughness parameters. In Table 3, the values of three roughness parameters (*R_a_*, *R_q_*, and *Rz*, see experimental section) are gathered. As can be seen, the quantitative results obtained through roughness confirm what is observed in the images. It seems, therefore, that the appearance of those protuberances causes the roughness variations. One-way ANOVA test showed there is a statistically significant difference (with α level 0.05) in the roughness represented by the parameters given in Table 3 when comparing the samples as a function of the concentration of chitosan.

**Figure 5:**
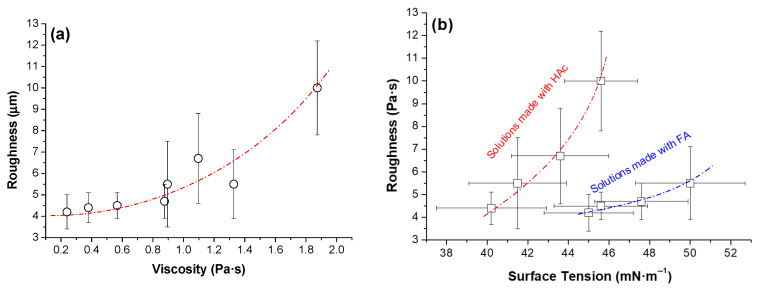
Roughness of AASS materials as a function of solutions’ viscosities (**a**) and surface tensions (**b**).

Both the viscosity and surface tension of the solutions sprayed seem to have a combined contribution to the roughness of the final materials. As the viscosity increases, there is a general trend for the roughness to increase (Figure 5a). This result makes sense if the flow of material when reaching the collector is more impeded and the corresponding spreading on the substrate surface is poorer. On the other hand, when surface tension is considered as the variable, the two sets of solutions must be looked at separately to notice the trend (Figure 5b). In both cases, the higher the surface tension, the higher the roughness. The higher the surface tension, the larger the droplets should be, so lower roughness might be expected, at least at the microscale. However, when the surface tension is considered without paying attention to the nature of the liquid, there is no trend, since the liquid with lower viscosity presents higher surface tension. Considering that both surface tension and viscosity interact in a non-linear manner, and their effects can depend on the atomization and evaporation regimes, we can only say that our results point out that viscosity appears to have a greater influence in this specific system, but to confirm the above further analysis—such as rheological measurements during spraying or computational fluid dynamics simulations (CFD simulations) would be necessary. On the other hand, the solvent evaporation rate and polymer–polymer interactions (such as possible formation of segregated chitosan domains) also can influence the morphology. Acetic acid and formic acid differ in volatility and polymer solubility, which can affect the redistribution of cellulose acetate (CA) and chitosan (CS) during droplet drying. Formic acid, being more volatile, promotes faster solvent loss, which can “freeze” local polymer concentrations before thorough mixing occurs, leading to more pronounced microphase segregation. This may result in core–shell or porous structures at the microscale, with CS potentially migrating towards the droplet surface. In contrast, the slower evaporation rate of acetic acid allows polymers to diffuse and rearrange within the droplet, producing a more homogeneous and continuous morphology upon deposition. The interplay between polymer concentration, viscosity, and evaporation kinetics further modulates this behavior, with higher viscosity or polymer loading limiting diffusion and enhancing phase segregation. Understanding these effects is essential for explaining the surface topography, porosity, and microstructural features of films produced by spraying and highlights the importance of solvent selection in controlling the morphology of polymer films prepared by AASS.

**Figure 6:**
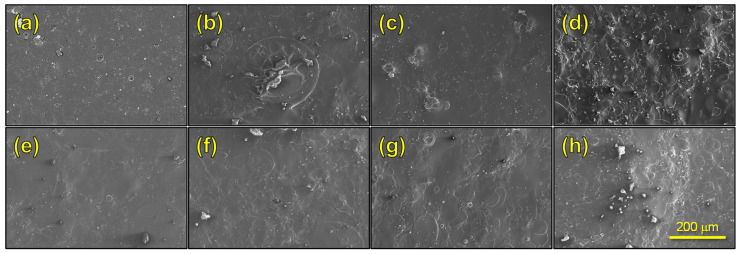
FESEM images of the top side of films produced by AASS, (**a**) HAc-CA; (**b**) HAc-CA/CS-1; (**c**) HAc-CA/CS-2; (**d**) HAc-CA/CS-3; (**e**) FA-CA; (**f**) FA-CA/CS-1; (**g**) FA-CA/CS-2; (**h**) FA-CA/CS-3.

To have higher lateral resolution, FESEM images were taken (Figure 6). It can be observed in all cases that a relatively flat surface whose topography is slightly irregular due to the presence of droplets of material that obtain flat by spreading out when they impact the collector during the spraying process. From the analysis of the microfeatures observed in the FESEM images, it is possible to confirm what was explained above about the contributions of viscosity and surface tension.

**Figure 7:**
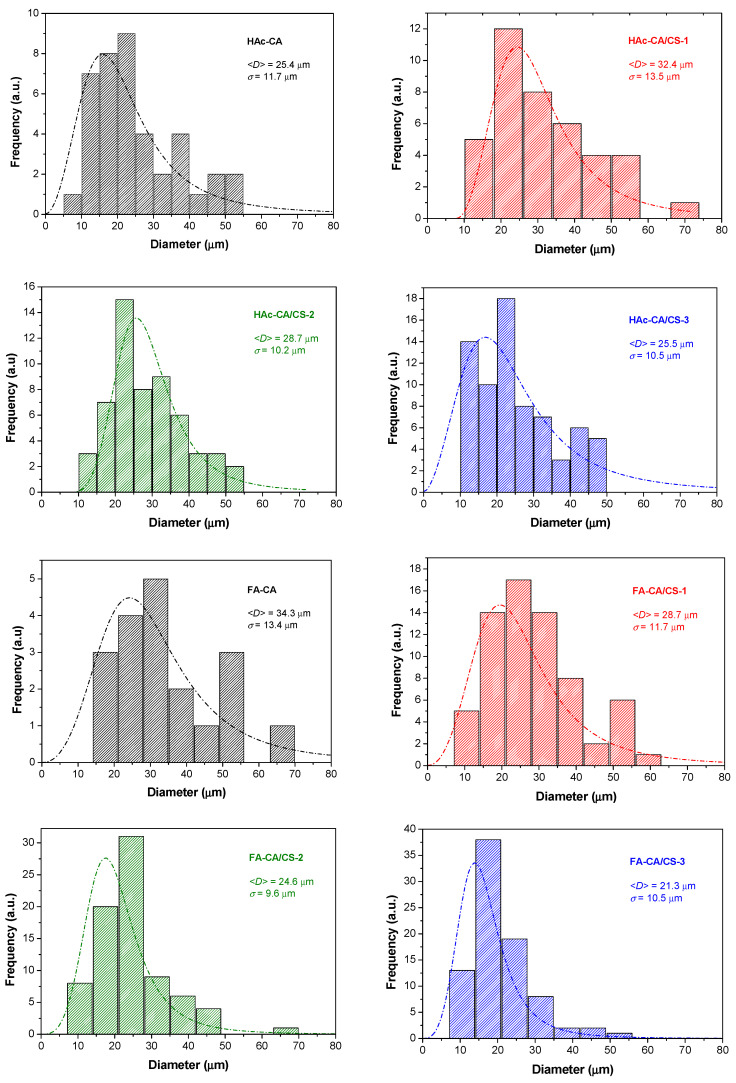
Size distribution of the droplets observed in the FESEM images of the materials analyzed by ImageJ software.

Using ImageJ, the size of droplets observed on the FESEM images was measured. In Figure 7, the distributions of droplet diameters are shown. This information should give a clue with respect to which contribution affects the final roughness of the materials the most. There is a clear tendency to decrease the size of droplets observed in the FESEM images as the viscosity and surface tension of the solution increase, i.e., chitosan concentration increases. For a set of samples made with a particular solvent, the lower the viscosity, the higher the ability for the droplet to spread out, and the lower the surface tension, the larger the droplets can be formed. Therefore, both parameters should contribute in the same direction in terms of the size of microfeatures observed. However, if both sets of samples are compared to each other in terms of materials with the same composition, the viscosity is the factor that conditions the most on the size of the microfeatures observed (Figure 6 and Figure 7). ANOVA test showed a statistically significant difference (with α level 0.05) in the mean values of droplet size between the samples that contain different amounts of chitosan.

**Figure 8:**
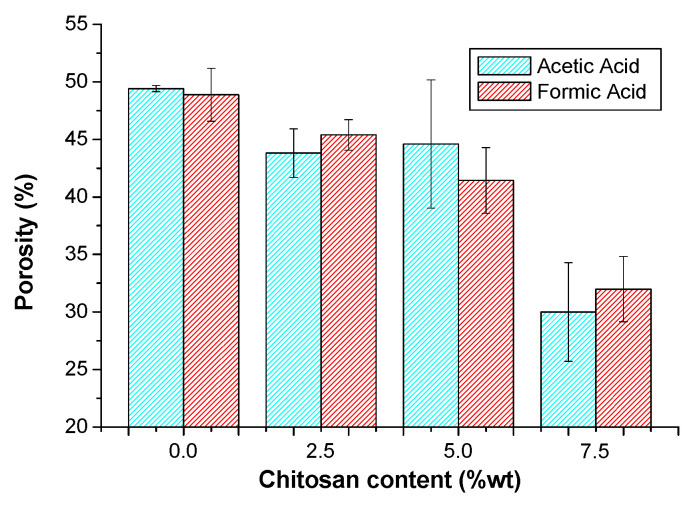
Porosities of all the samples prepared in this study.

Additionally, to better understand surface properties of the materials, such as solvophilicity behavior, water vapor permeability, and bioactivity, porosity was also analyzed. Figure 8 shows the porosities of all the samples prepared in this study. As can be seen, all materials are quite porous. For a particular composition, there is no clear difference in porosity as a function of the solvent used. However, what is observed is a clear trend to decrease the porosity when the concentration of CS increases, which implies an increase in the surface tension. A possible explanation can be a combination of the viscosity and surface tension increase in the solutions to be sprayed. Although the increase in viscosity must oppose the rapid spreading of the drops on the collector surface, an increase in surface tension must favor a larger droplet size that allows for greater coating efficiency and, consequently, lower porosity. However, it is important here to highlight that the presence of CS may also increase packing density and reduce porosity due to a filling effect or improved matrix cohesion; therefore, porosity reduction could be considered multifactorial, involving increased structural cohesion, reduced spreading ability, and higher packing density of CS-rich droplets.

### 3.3. Solvophilicity Behavior

**Figure 9:**
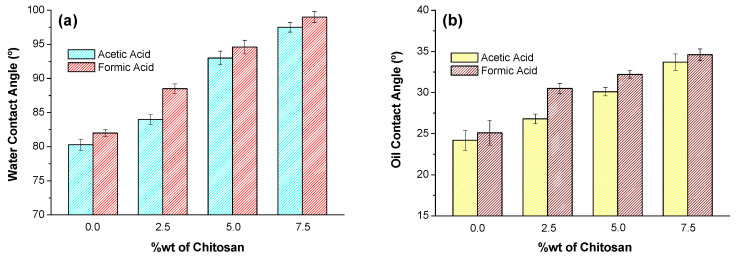
(**a**) Water and (**b**) oil contact angles of droplets on the CA-based materials with different concentrations of chitosan, depending on the solvent used for material preparation, using AASS.

Once the morphology is known in terms of the size and shape of the microfeatures observed together with the porosity, the ability of the materials to be wetted by liquids such as water and oil can be interpreted. Water and oil contact angles were measured, and the corresponding values are represented in Figure 9.

When analyzing contact angles, two contributions must be taken into consideration: physico-chemical interactions and topography. The first contribution should be associated with the nature of the material, and the higher the polarity, the higher the hydrophilicity, which should correspond to a lower water contact angle and higher oil contact angle, respectively. For the materials under consideration, the nature changes as a function of the composition or the concentration of CS. The value of surface tension published for pellets of chitosan with Mw = 58,000 Da and DDA = 67% is 31 J·m^−2^ with only dispersive contribution [60] while values of around 50 J·m^−2^ were reported for cellulose acetate, where the polar contribution was 17 J·m^−2^ [61]. Therefore, with respect to the surface, the higher the proportion of CA, the higher the hydrophilicity is expected, considering reported data or the simple application of the rule of mixtures [62]. However, the results shown in Figure 9 indicate that both hydrophobicity and oleophobicity increase with higher proportions of CS, which appears to be contradictory. The observed behavior reflects the influence of surface morphology, where increased CS content affects both water and oil contact angles similarly due to changes in porosity and roughness, rather than implying that hydrophilicity and hydrophobicity increase simultaneously in a literal sense. In other words, these results can only be explained through the consideration of morphology as the main contribution to the ability of liquid droplets to spread out on a surface. Then, following the general trends observed, the porosity in combination with roughness should be the parameters that correlate with the contact angles, regardless of the nature of the liquid. What can be seen is that there must be a balance between both parameters since the lower the porosity (Figure 8) and the higher the roughness (Table 3), the higher the contact angle, regardless of the nature of the liquid. However, when comparing materials with the same concentration of CS but prepared with different solvents, an apparent contradiction is observed since, for almost the same porosity (Figure 8), the contact angles are slightly higher (if error is not considered) when the roughness is lower (Figure 9). A possible explanation of this result is to consider the porosity as the parameter that contributes the most to the wettability. The observed contact angles can be explained using the Wenzel [63] and Cassie-Baxter [64] models, which describe how surface morphology influences wettability. While chemical composition suggests that increasing cellulose acetate (CA) should enhance hydrophilicity, our results show that both hydrophilicity and hydrophobicity increase with higher chitosan (CS) content. This can be attributed to the dominant role of surface morphology: porosity controls air entrapment and droplet spreading (Cassie-Baxter effect), while roughness amplifies the intrinsic wettability (Wenzel effect). Thus, the interplay between porosity and roughness, rather than chemical composition alone, primarily determines the observed contact angles.

### 3.4. Water Vapor Permeability

**Figure 10:**
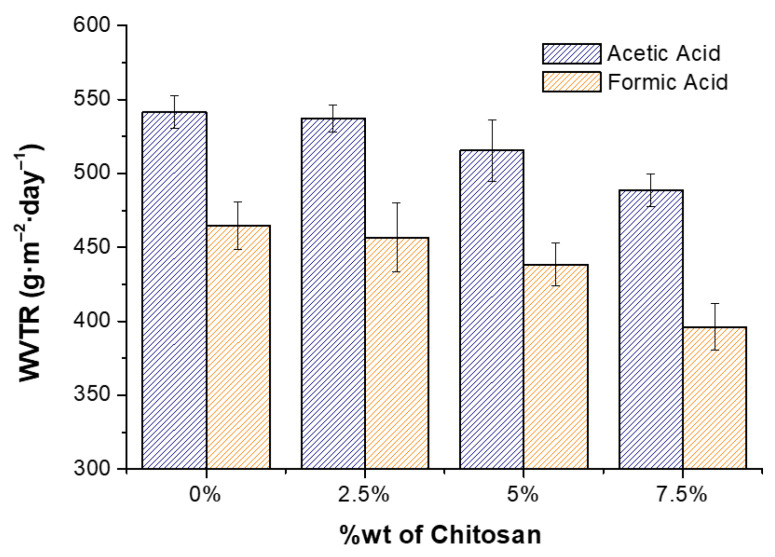
Values of water vapor transmission rate (WVTR) for CA-based materials with varying chitosan concentrations.

The water vapor transmission rate is a critical property in food packaging because it prevents moisture transfer, helping to maintain the ideal moisture content inside the packaging. In Figure 10, the values of WVTR obtained for the different materials under study are presented.

As can be seen, the WVTR of all the samples is significantly lower than previously reported for some cellulose acetate-based films [65]. As the concentration of chitosan increases in the composite, the WVTR further decreases. This supports the notion that polar groups in components of composite films can be involved in intra- or intermolecular interactions (e.g., hydrogen bonding) that reduce their availability to interact with water, thus creating a water barrier material. The values of WVTR obtained in this work are significantly lower (550 g·m^−2^ day^−1^), compared with previously reported values for cellulose acetate, and are of the same magnitude as grafted cellulose acetate [66]. In addition, when comparing the samples prepared from solutions made with the different solvents, it is observed that when using FA as the solvent, WVTR is lower, which further supports the hypothesis that lower porosity decreases the ability of the material to transfer water vapor through it.

Jointly observing the results of wettability and water vapor transmission, it can be concluded that the combination of two moderately hydrophilic polymers through processing with solution blowing and creating a specific morphology may lead to obtaining flexible and sustainable materials with properties applicable for food packaging. However, it is important here to recall that moisture sensitivity is a property that should be considered depending on the final application. In certain applications, it is preferred to have higher moisture sensitivity, for example, when using multilayer systems where one of the layers is more moisture sensitive but covered by hydrophobic coatings to regulate vapor transfer. Other examples are cellulose acetate films modified with hydrophilic polymers or salts that absorb moisture, trying to delay mold growth in baked goods. Also, water-soluble or at least partially soluble polymers could be interesting for controlled release systems and edible or biodegradable coatings.

### 3.5. Mechanical Behavior of Films

**Figure 11:**
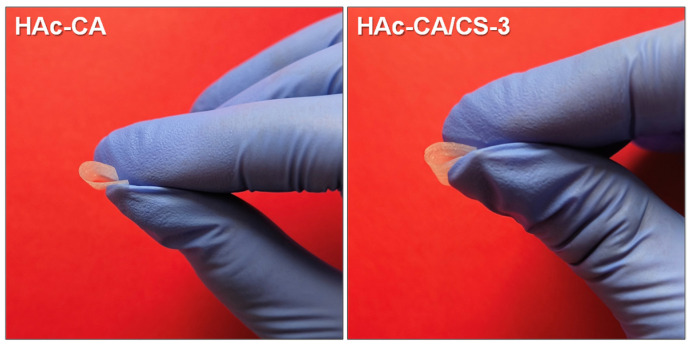
Representative images of the materials prepared, showing their high flexibility.

Mechanical behavior is another important aspect to control during materials preparation, especially considering the final application. For food packaging, the materials are required to be mechanically consistent but also flexible. Figure 11 shows two representative images of the materials prepared. It is seen that all materials present a high flexibility. Cellulose acetate can be either flexible or brittle depending on several factors, primarily its degree of substitution, the presence of plasticizers, and the processing method. For example, when the degree of substitution is higher than 2.4, as in our case, without plasticizers, films of CA are rigid and brittle. In addition, films obtained by casting or slow drying tend to be more brittle if they do not contain plasticizers. Therefore, the flexible behavior shown by the materials prepared by AASS indicates they are good candidates to be used for food packaging.

On the other hand, all materials in this work were tensile tested (Figure S3) to obtain the corresponding strength–strain curves from which values of Young’s modulus (*E*), maximum strength (*σ_max_*), and strain at failure (ε_f_) were determined. In Table 4, all those mechanical parameters are presented. A one-way ANOVA test showed there is a statistically significant difference (with α level 0.05) in all the mechanical parameters considered in Table 4 when comparing the samples as a function of the concentration of chitosan or when comparing between materials obtained with different solvents.

As can be seen, both the solvent used to prepare the solutions to be sprayed and the content of chitosan influence the mechanical behavior. Regardless of the solvent used, there is a general trend of increasing the maximum strength and the strain at break of the material by the addition of chitosan, while there is a decrease in the Young’s modulus. When acetic acid is used as a solvent, there is an outstanding increase in the maximum strength and strain of the film with the highest chitosan content (7.5%), almost 3 times in the first case and 4 times in the second, compared to the neat cellulose acetate film produced from acetic acid. A similar trend is observed when formic acid is used as the solvent to prepare the materials.

To explain these results, a balance between two contributions can be considered: porosity (Figure 8) and crystallinity degree (Table 2). In principle, the first one should account more for the tensile strength since lower porosity yields more effective cross-sectional area supporting the mechanical load, which in turn should lead to higher tensile strength. On the other hand, the higher the crystallinity degree, the higher the modulus, which may influence the tensile strength. In fact, the highest moduli are found for the samples with the highest crystallinity, which are those prepared by using formic acid as solvent. Despite the above, it is important to highlight the fact that in this work, solution spraying was able to produce flexible and stretchable materials. This behavior may be attributed to the specific microstructure generated by the solution spraying process, which promotes a more homogeneous stress distribution throughout the film and may introduce a fine porous or fibrillar network. Such morphological features reduce brittleness and enable the material to deform without fracturing, thereby enhancing flexibility and stretchability. These properties are crucial for food packaging materials; therefore, the SBS is an excellent choice for flexible film preparation, especially from rigid carbohydrates without the use of any plasticizer.

Here, It Is Important to point out that water content can sometimes Influence the final mechanical behavior under certain conditions; however, the specimens in this work were stored under the same conditions, and no differences were observed in the FTIR spectra in terms of water content (Figure 2). The results obtained in the present study revealed that if all samples have absorbed water, it must be negligible. It is well-known that very small amount of absorbed water in cellulose acetate causes subtle but detectable changes in the IR spectrum such as increase in intensity and broadening of the OH band (3200–3600 cm^−1^), slight decrease in the ester C=O peak (~1745 cm^−1^), and minor alterations in the C–O–C band of the acetate (~1230 cm^−1^). These variations are useful in ATR-FTIR spectroscopy for monitoring moisture changes in cellulose acetate. However, the FTIR spectra obtained for the CA-based materials prepared in the present work do not present those alterations mentioned. As an example, to confirm the latter, Chris Toprak et al. studied the water absorption of cellulose acetate with a degree of acetylation of 2.45 using FTIR in the transmission mode [66]. They found that with a very small amount of water, it is possible to observe clear changes in the absorbance of the bands mentioned above. On the other hand, Murphy et al. [67], using cellulose acetate membranes with the same degree of acetylation and similar molecular weight to the CA used in the present work, reported even higher relative absorbance (obtained by ATR-FITR) of the broad band corresponding to OH stretching for dry samples subjected to a process based on the use of isopropanol to remove the water. As a conclusion, and considering the result obtained from ATR-FTIR spectra in the present work, the water content in the samples under study can be considered negligible, and, as a consequence, no effect should be expected in their mechanical performance.

Finally, it is also important to make a comparison with similar systems obtained by other, more conventional methods, such as solvent casting. For example, A.W. Cindradewi et al. prepared by solvent casting and characterized cellulose acetate films reinforced with cellulose nanofibrils [68]. They obtained for the pure cellulose acetate with a similar degree of acetylation, a tensile strength (80 Mpa), Young’s modulus (4 Gpa), and elongation at break (5%), higher than 90%, higher than 80%, and lower 70%, respectively. The main reason for these results must be the porosity, since in the case of solvent casting, it is expected to obtain perfect bulk materials if the rate of evaporation is well controlled. The values of tensile strength and Young’s modulus are conditioned by the section area considered to obtain the tensile test curves and, consequently, the mechanical parameters. For porous materials, the real section area that supports the mechanical loads is replaced by the higher section area directly obtained from the measured size of the specimen (width and thickness). However, the important thing here is that enough mechanical consistency is attained by producing the materials by AASS, and that enhanced flexibility is achieved.

### 3.6. Antimicrobial Activity Against Gram-Negative E. coli

**Figure 12:**
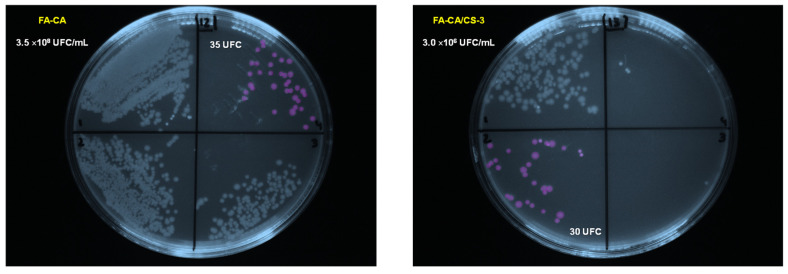
Images of bacterial colonies produced in the samples produced from solutions made with formic acid, without (**left**) and with 7.5% of chitosan (**right**).

Based on previous results presented here, the sample produced from a solution made with formic acid and with 7.5% chitosan can be considered the one with satisfactory properties (mechanical, water barrier, and wettability) to be considered the most appropriate for food packaging applications. Therefore, this sample was selected to study its bioactivity against *E. coli*. Figure 12 shows images of bacterial colonies produced in the corresponding samples processed from formic acid using solution spraying without and with 7.5% of chitosan. The analysis revealed that the number of colonies in the cellulose acetate/chitosan blend is significantly reduced (~2 orders of magnitude lower), indicating a potential antibacterial effect of the material on food preservation. These results testify that the antimicrobial activity of chitosan was retained in the composite matrix, likely due to partial availability of its functional groups, for example, the positively charged amino groups that could interact with the bacterial cell wall [69,70]. This result is also expected based on the fact that the antimicrobial activity of chitosan is frequently reported to be mostly pronounced towards Gram-negative bacteria [71]. This is a consequence of the interaction between positively charged amino groups of chitosan and the negatively charged cell wall of bacteria. If we consider this aspect and accept the possibility of interaction between amino groups of chitosan, we can confirm that in the composite developed in this work, interactions between components are mostly based on OH groups between cellulose acetate and chitosan.

## 4. Conclusions

In this work, the preparation of innovative blends in the form of films from cellulose acetate and chitosan using air-assisted solution spraying (AASS) is presented. Films developed by AASS have a unique morphology consisting of droplets that merge during production, outstanding mechanical properties (up to 80 MPa maximum strength depending on the blend composition and solvent used for materials preparation), high contact angle towards water (up to 95°), and moderate contact angle with oil (<90°). Mentioned properties, taken together with antibacterial activity against E. coli, a Gram-negative bacterium, make these films the perfect candidates for food packaging applications. Considering the increasing need to obtain sustainable materials while decreasing carbon footprint and negative environmental impact, the processing of biopolymers with solution blow spinning represents a significant step toward sustainability goals. The absence of any plasticizer in the formulation while maintaining the flexibility and mechanical integrity of films is a great advantage since the presence of plasticizers is frequently of short duration, and their leaching from the material prevents this kind of material from being used for food packaging. The mechanical and sorption properties, together with bioactivity of developed materials, alongside ease of processing using AASS, testify to the superiority of this technique over others for film preparation (casting, molding) and bring high potential into the sector of food packaging materials. Although the purpose of the present work was not the direct deposition of materials on the food, the information gathered in this study should be potentially useful when understanding the performance of these materials if they are directly deposited by AASS on any target, including food items for their preservation.

## Figures and Tables

**Table 1:** Values of viscosities and surface tensions of all solutions prepared to be sprayed are collected.

System	Viscosity (Pa·s)	Surface Tension (mN·m^−1^)
HAc-CA	0.379 ± 0.031	40.2 ± 2.7
HAc-CA/CS-1	0.895 ± 0.073	41.5 ± 2.4
HAc-CA/CS-2	1.096 ± 0.086	43.6 ± 2.4
HAc-CA/CS-3	1.873 ± 0.145	45.6 ± 1.8
FA-CA	0.239 ± 0.021	45.0 ± 2.2
FA-CA/CS-1	0.567 ± 0.046	45.6 ± 2.3
FA-CA/CS-2	0.876 ± 0.071	47.6 ± 2.3
FA-CA/CS-3	1.328 ± 0.109	50.0 ± 2.7

**Table 2:** Values of glass transition temperature, *T_g_*, melting temperature, *T_m_*, and degree of crystallinity, *χ*, of the CA-based materials.

Sample	*T_g_* (°C)	*T_m_* (°C)	*χ* (%)
HAc-CA	197	226	6.0
HAc-CA/CS-1	198	227	6.5
HAc-CA/CS-2	198	228	5.0
HAc-CA/CS-3	-	229	7.5
FA-CA	-	229	15.3
FA-CA/CS-1	-	229	13.6
FA-CA/CS-2	-	229	11.5
FA-CA/CS-3	-	231	11.2

**Table 3:** The roughness parameters of the CA-based materials obtained by AASS.

Sample	*R_a_* (µm)	*R_q_* (µm)	*R_z_* (µm)
HAc-CA	4.4 ± 0.7	5.9 ± 1.1	34.4 ± 9.5
HAc-CA/CS-1	5.5 ± 2.0	7.0 ± 2.4	36.1 ± 8.7
HAc-CA/CS-2	6.7 ± 2.1	8.5 ± 2.7	41.1 ± 16.4
HAc-CA/CS-3	10.0 ± 2.2	12.4 ± 2.2	58.8 ± 10.6
FA-CA	4.2 ± 0.8	5.4 ± 1.0	29.2 ± 6.1
FA-CA/CS-1	4.5 ± 0.6	5.7 ± 0.9	29.4 ± 7.0
FA-CA/CS-2	4.7 ± 0.8	5.9 ± 1.0	29.5 ± 5.7
FA-CA/CS-3	5.5 ± 1.6	7.0 ± 1.9	34.3 ± 7.7

**Table 4:** Mechanical parameters obtained from the tensile tests of the CA-based materials prepared by AASS.

Sample	*E* (Mpa)	*σ*_max_ (Mpa)	ε_f_ (%)
Hac-CA	626 ± 14	27 ± 2	4.9 ± 0.5
Hac-CA/CS-1	545 ± 15	43 ± 6	10.3 ± 0.7
Hac-CA/CS-2	482 ± 55	56 ± 3	17.6 ± 1.8
Hac-CA/CS-3	467 ± 21	80 ± 1	19.3 ± 0.8
FA-CA	946 ± 61	19 ± 3	2.1 ± 0.2
FA-CA/CS-1	633 ± 10	37 ± 2	9.9 ± 0.5
FA-CA/CS-2	619 ± 38	44 ± 1	10.2 ± 0.5
FA-CA/CS-3	538 ± 50	64 ± 5	12.9 ± 1.3

## Data Availability

All data will be available upon request.

## Supplementary Materials

The following supporting information can be downloaded at: https://www.mdpi.com/article/10.3390/polym17182479/s1, Table S1: Solubility data for the cellulose acetate (CA), chitosan (CS), and mixtures CA/CS; Figure S1: Representative photos of the CA-based films prepared by solution spraying; Figure S2: X-ray diffraction patterns of the as-received polymers CA and CS, respectively; Figure S3: Stress–strain curves obtained from tensile tests performed for all the specimens of each sample.
