# Supplementary material for: Air-Assisted Sprayed Flexible Cellulose Acetate/Chitosan Materials for Food Packaging
Source: Polymers (Basel). 2025 Sep 13;17(18):2479. doi: 10.3390/polym17182479 (PMC12473453; doi:10.3390/polym17182479)
Supplement: Supplementary file 1 [file polymers-17-02479-s001.zip › polymers-3867946-supplementary.pdf]

Supplementary material

Air-assisted sprayed flexible cellulose acetate/chitosan materials for food packaging

Nasrin Moshfeghi Far [1], Ana Kramar [2] and Javier González-Benito [1,*]

[1] Department of Materials Science and Engineering and Chemical Engineering, Universidad Carlos III de Madrid, Av. Universidad 30, 28911 Leganés, Spain

[2] Novel Materials and Nanotechnology Group, Institute of Agrochemistry and Food Technology (IATA), Spanish Council for Scientific Research (CSIC), Calle Catedrático Agustín Escardino Benlloch 7, 46980 Paterna, Spain; akramar@iata.csic.es

* Correspondence: javid@ing.uc3m.es; Tel.: +34 91624948870

Solubility study

To find out the best conditions to process the polymer systems through solution blow spinning or spraying, it is necessary to choose an adequate solvent that could properly dissolve the biopolymers, giving the solution the most adequate properties in terms of viscosity, surface tension, and evaporation rate. In order to find the most appropriate conditions to prepare solutions of the polymers under study, together with a mixture of them, trial-and-error tests were conducted using different solvents and mixtures of solvents. A small amount of the polymer system (~ 20 mg) was added to a vial, followed by a particular solvent system (~ 2 mL). After waiting for a certain time, the vials were visually inspected, looking for the clarity of the solution, which was interpreted as evidence of proper dissolution. In all cases, the temperature was increased to check if solubility could be induced or accelerated. Two possible results can be obtained: a clear solution (positive, +) and a non-clear solution (negative, -). Table S1 summarizes the corresponding qualitative results obtained.

It can be seen that two solvent systems can be used to properly dissolve CA when combined with chitosan with a relatively low amount of CS. The two solvent systems are acetic acid (HAc) and water in a proportion 9:1 and formic acid (FA). Therefore, these two solvents were chosen to prepare the corresponding solutions to be sprayed. HAc:H_2O (9:1) at 60 ºC and FA at 25 ºC.

Table S1. Solubility data for the cellulose acetate (CA), chitosan (CS), and mixtures CA/CS.

Polymer system	Concentration (% wt)	Solvent	Conditions	Result
CA	1	Acetic Acid	40min/stirring/25ºC	+
CA	10, 12, 14 and 16	THF	1h-3h/stirring/25ºC	+
CA	1	DMF	20min/stirring/25ºC	+
CA	1	Formic Acid	3h/stirring/25ºC	+
CA	10	Formic Acid	3h/stirring/25ºC	+
CA	1	Acetonitrile	1h/stirring/25ºC	+
CS/CA	1/3	Formic Acid	3h/stirring/25ºC	+
CS/CA	5/10	Formic Acid/ Acetic Acid (1:1)	3h/stirring/25ºC	+ (high viscosity)
CS/CA	0.5/10	Acetic Acid: H_2O (9:1)	2h/stirring/60ºC	+
CS/CA	1/10	Acetic Acid: H_2O (9:1)	8h/stirring/60ºC	+ (High viscosity)
CS/CA	0.5/8	Acetic Acid0.1M: Acetone (1:1)	2h/stirring/25ºC	-
CS/CA	0.5/8	Formic Acid	3h/stirring/25ºC	+
CS/CA	0.5/8	Acetic Acid: H_2O (9:1)	2h/stirring/60ºC	+
CS	5	THF	24h/stirring/25ºC	-
CS	3	THF	24h/stirring/25ºC	-
CS	1	THF	24h/stirring/25ºC	-
CS	1	Acetonitrile	24h/stirring/25ºC	-
CS	1	Formic Acid	4h/stirring/25ºC	+
CS	3	Formic Acid	5h/stirring/25ºC	+
CS	1	DMF	24h/stirring/40ºC	-
CS	0.1	DMF	24h/stirring/40ºC	-
CS	1	Acetic Acid	24h/stirring/40ºC	-
CS	1	Acetic Acid 0.1M	5h/stirring/60ºC	+

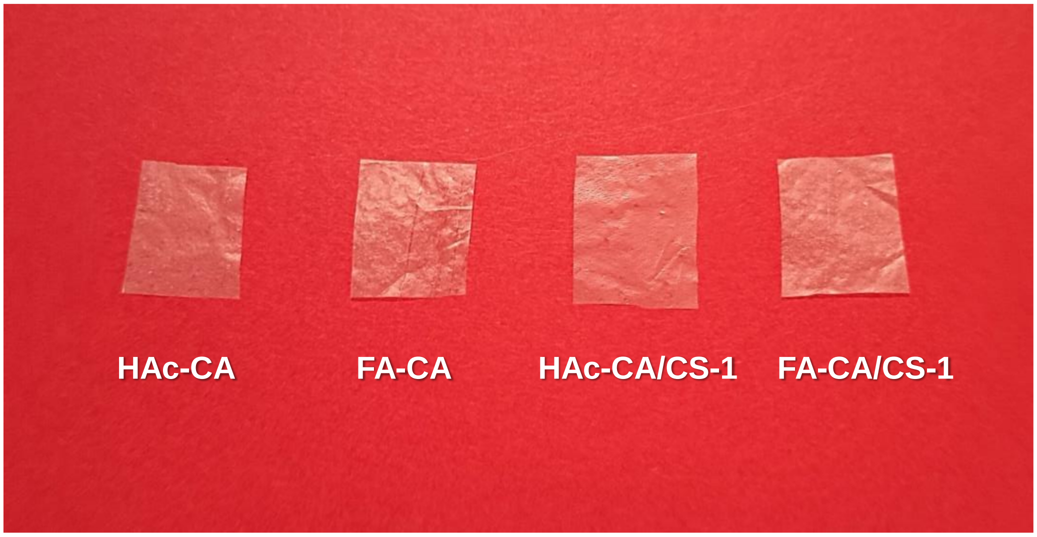

Figure S1. Representative photos of the CA-based films prepared by solution spraying.

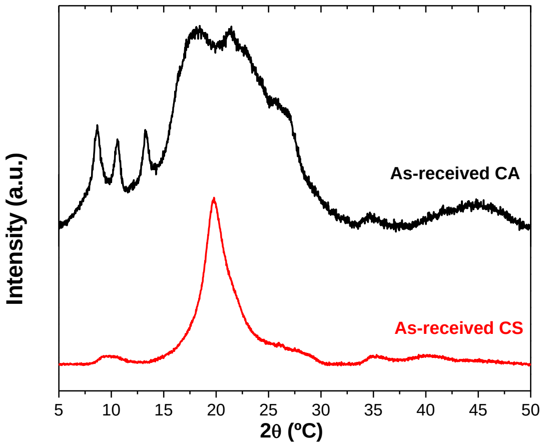

Figure S2. X-ray diffraction patterns of the as-received polymers CA and CS, respectively.

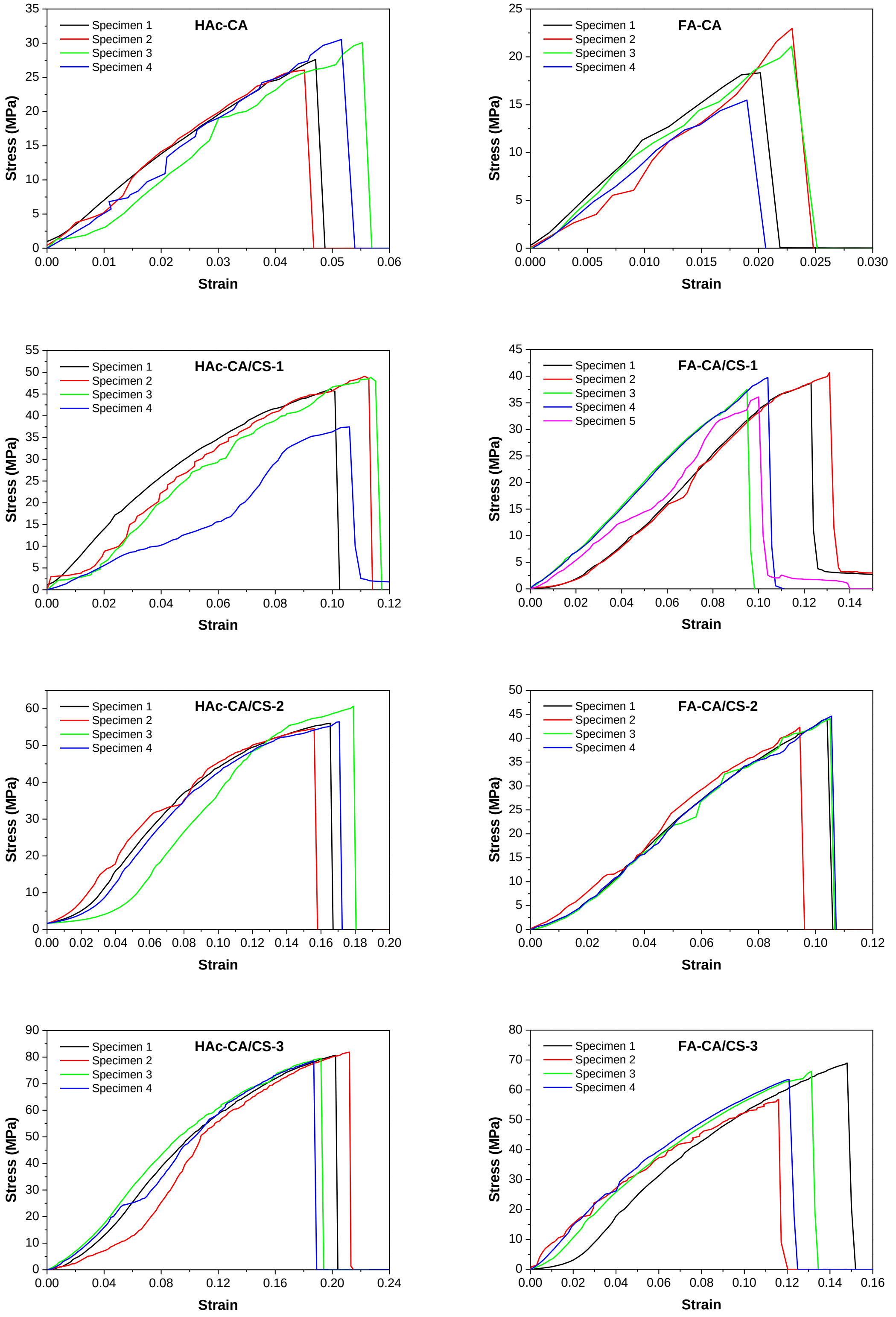

Figure S3. Stress-strain curves obtained from tensile tests performed for all the specimens of each sample.
